# Maternal breast cancer risk in relation to birthweight and gestation of her offspring

**DOI:** 10.1186/s13058-018-1035-6

**Published:** 2018-10-05

**Authors:** Anthony J. Swerdlow, Lauren B. Wright, Minouk J. Schoemaker, Michael E. Jones

**Affiliations:** 1 Division of Genetics and Epidemiology, The Institute of Cancer Research, Sir Richard Doll Building, London, SM2 5NG UK; 2 Division of Breast Cancer Research, The Institute of Cancer Research, London, SW3 6JB UK

**Keywords:** Breast cancer, Risk, Birthweight, Gestation, Offspring

## Abstract

**Background:**

Parity and age at first pregnancy are well-established risk factors for breast cancer, but the effects of other characteristics of pregnancies are uncertain and the literature is inconsistent.

**Methods:**

In a cohort of 83,451 parous women from the general population of the UK, which collected detailed information on each pregnancy and a wide range of potential confounders, we investigated the associations of length of gestation and birthweight of offspring in a woman’s pregnancies with her breast cancer risk, adjusting for a full range of non-reproductive as well as reproductive risk factors unlike in previous large studies.

**Results:**

Gestation of the first-born offspring was significantly inversely related to the risk of pre-menopausal breast cancer (*p* trend = 0.03; hazard ratio (HR) for 26–31 compared with 40–41 weeks, the baseline group, = 2.38, 95% confidence interval (CI) 1.26–4.49), and was borderline significantly related to risk of breast cancer overall (*p* trend = 0.05). Risk was significantly raised in mothers of high birthweight first-born (HR for breast cancer overall = 1.53, 95% CI 1.06–2.21 for ≥ 4500 g compared with 3000–3499 g, the baseline group). For gestation and birthweight of most recent birth, there were no clear effects. Analyses without adjustment for confounders (other than age) gave similar results.

**Conclusions:**

Our data add to evidence that short gestation pregnancies may increase the risk of breast cancer, at least pre-menopausally, perhaps by hormonal stimulation and breast proliferation early in pregnancy without the opportunity for the differentiation that occurs in late pregnancy. High birthweight first pregnancies may increase breast cancer risk, possibly through the association of birthweight with oestrogen and insulin-like growth factor 1 levels.

**Electronic supplementary material:**

The online version of this article (10.1186/s13058-018-1035-6) contains supplementary material, which is available to authorized users.

## Background

It has long been known that a woman’s risk of breast cancer relates to the number of pregnancies she has had and the age at which she first gave birth [1, 2]. Serum levels of oestrogens and several other hormones are far higher during pregnancy than at other points in life [3–5], and there are major histological changes in the breast during pregnancy [6] giving plausible mechanisms for such effects. One might therefore expect that characteristics of the pregnancies, such as the length of gestation and the birthweight of the offspring, that are associated with maternal hormone levels [7–14] might also affect breast cancer risk. Investigations of this have been far more limited, however, than for age at first birth and parity, and results have been inconsistent. The large studies [15–24] have all been based on record linkage, and consequently have generally not been able to adjust for non-reproductive confounders, which might be important since several factors such as maternal height, birthweight, smoking, and alcohol consumption, that are associated with the offspring’s birthweight and gestation [25–31], are also known or likely risk factors for breast cancer. Also, with one exception for hormone receptor type [23], these studies have not analysed risks in subdivisions of breast cancer that may have different aetiological characteristics—by menopausal status at the time of breast cancer incidence, by in-situ versus invasive status, and by hormone receptor subtype.

We therefore analysed the relation of offspring’s birthweight and gestation to maternal breast cancer risk in a large UK cohort in which information on birthweight and other characteristics was collected separately for each pregnancy, and in which extensive information was collected on potential confounders and virtually complete information on breast cancer characteristics.

## Methods

The Generations Study is a cohort study of > 113,000 women from the general population of the UK recruited since 2003 at age 16 years and older [32]. At recruitment, the participants completed a detailed questionnaire about potential risk factors for breast cancer and donated a blood sample. The recruitment questionnaire asked about several variables, including birthweight and gestation, the variables addressed in this paper, for each of the subject’s pregnancies, and also about all known and likely causes of breast cancer. The pregnancy information was updated at the second follow-up questionnaire, 6 years after recruitment, for any subsequent pregnancies.

Follow-up of the cohort is primarily by questionnaires, which have been completed at approximately 3-year intervals with high completeness: by 99% of non-deceased participants at the first follow-up, 97% at the second, and 95.5% at the third. Follow-up for cancer incidence and mortality of the small proportion who did not respond to questionnaires was by ‘flagging’ at the National Health Service Central Registers (NHSCRs), virtually complete population registers of England and Wales, and of Scotland, in which deaths and cancer registrations are ‘flagged’ and are then reported to authorised medical researchers. The study was approved by the South East Multi-centre Ethics Committee.

Cancers occurring in the cohort during follow-up were identified from follow-up questionnaire responses and from spontaneous reports to the investigators, as well as by flagging at the NHSCRs of participants who did not reply to questionnaires. Cancer diagnoses were confirmed by cancer registration data via the NHSCRs, pathology reports, and correspondence with physicians. To facilitate comparison with the great majority of the previous literature [15–19, 22–24, 33, 34], we have presented analyses for invasive breast cancer in the main tables, but also have analysed risks for in-situ breast cancer (in Additional file 1:Table S1) for which there appear to be no previous data.

The current analytic cohort consisted of all women who were recruited into the Generations Study from its launch (June 2003) to December 2013, without prior invasive breast cancer or mastectomy. The cut-off date of December 2013 was used because, at the time of analysis, at least second-round follow-up was complete for these recruits (timing of follow-up rounds is personalised depending on their date of recruitment).

We initially analysed first-birth characteristics in relation to subsequent risk of breast cancer. For this, study subjects entered analytic risk at the date they were recruited to the cohort if already parous, or at the date of first delivery at ≥ 26 weeks gestation if recruited nulliparous, and left at the earliest of first invasive breast cancer diagnosis, death, most recent follow-up questionnaire, or loss to follow-up. Women whose first delivery was of twins or multiple babies were excluded from these analyses since having borne twins has been found to be associated with a reduced risk of breast cancer [35] and twins tend to be delivered early and have reduced birthweight. Similar analyses were conducted for risks in relation to most recent (‘last’) ≥ 26 weeks delivery on a time-dependent basis (i.e. re-setting duration since last birth to zero after each birth).

We also conducted separate analyses of follow-up before and after 15 years since delivery, since there is strong evidence that pregnancy has two opposite effects on breast cancer risk: an increase in the short-term, up to about 15 years, followed by a long-term protective effect [36].

We estimated hazard ratios (HRs) and 95% confidence intervals (CIs) for risk of breast cancer in relation to gestation and birthweight of offspring, by left-truncated right-censored Cox proportional hazards regression [37] with attained age as the implicit time scale. Analyses were adjusted for multiple reproductive and non-reproductive potential confounders, as listed in the footnotes to the Tables. Time since entry to cohort, smoking start age, alcohol consumption, current oral contraceptive use, parity, menopausal status, and menopausal hormone therapy use, were treated as time-varying exposures. All *p* values are two-sided. Analyses were conducted using Stata, version 14.2 [38].

Trends were analysed in the Cox regression model by fitting continuous values of the risk factor, and subjects with missing data for the risk factor were included in the statistical model as a separate categorical term by fitting appropriate interaction terms. Tests for trend were determined by assessing whether the slope parameter in the statistical model differed from zero using the Wald test [39].

Tests for interaction in trends for analyses by menopausal status (to assess effect modification) were assessed by fitting additional interaction terms for menopausal status and the continuous risk factor and testing if the difference in trends differed from zero using the Wald test. For analyses by subtype of breast cancer defined by receptor status (to assess effect heterogeneity) we used a data augmentation method [40] to test if the difference in trends differed from zero using the Wald test.

Since pre-eclampsia has been found to be associated with reduced risks of subsequent breast cancer [41] and often with early delivery and reduced birthweight, and diabetes is associated with breast cancer risk (although only post-menopausally) [42, 43] and raised birthweight of offspring [44], we conducted sensitivity analyses excluding women with diabetes and women with pre-eclampsia in the birth(s) under analysis.

## Results

There are 107,480 participants in the Generations Study cohort recruited in 2003–2013 who did not have prior breast cancer or mastectomy at recruitment, of whom 85,069 had had any pregnancies by the time of their second follow-up questionnaire and formed the potential analytic cohort.

For analyses of first births, we excluded 1214 women whose first birth was twins or a higher order, and 404 with inadequate or erroneous data, leaving 83,451 for analysis. For analyses of other births there were similar exclusions. The characteristics at recruitment of the analytic cohort are shown in Table 1. Women entered the cohort at a wide range of ages, largely under 65 years, and half had had two pregnancies before recruitment.

**Table 1:** Characteristics of analytic cohort

Characteristic	*n*	%
Year of birth		
1908–39	5899	7.1
1940–49	20,519	24.6
1950–59	21,619	25.9
1960–69	19,350	23.2
1970–96	16,064	19.2
Year of start of analytic follow-up^a^		
2003–5	24,015	28.8
2006–7	39,062	46.8
2008–13	20,374	24.4
Age at start of analytic follow-up (years)^a^		
16–34	11,950	14.3
35–44	19,741	23.7
45–54	21,016	25.2
55–64	22,461	26.9
≥ 65	8283	9.9
Parity at start of analytic follow-up^a^		
1	19,917	23.9
2	42,862	51.4
3	16,229	19.5
≥ 4	4443	5.3
Total	83,451	100.0

^a^Original recruitment (entry) to the cohort for most subjects (*n* = 76,436), but date of first delivery at ≥ 26 weeks gestation for those who were nulliparous at original cohort entry (*n* = 7015)

During follow-up after first birth, 980 subjects had died, 1767 had developed invasive breast cancer, 80,043 had survived to their end date without breast cancer, and 661 were lost to follow-up (e.g. because of emigration) before their end date. We obtained pathological confirmation of diagnoses for all of the reported breast cancers except 12, which were based on self-report plus a report of appropriate treatment.

Table 2 shows the risks of breast cancer in the cohort in relation to birthweight and gestation of the offspring of the woman’s first pregnancy, overall and by menopausal status at breast cancer. Compared with mothers of offspring with birthweights 3000–3499 g, the most numerous group, risk was significantly raised (HR = 1.53, 95% CI 1.06–2.21) in those with the heaviest offspring (≥ 4500 g), with non-significant tendencies in the same direction for pre-menopausal and post-menopausal breast cancer separately. There was also a tendency to raised risks for mothers of the lowest birthweight children, which was significant for pre-menopausal but not for overall breast cancer. For gestation, there was a borderline significant inverse trend in breast cancer risk overall (*p* = 0.05), which was significant pre-menopausally (*p* = 0.03), with the greatest risk for those who had given birth at 26–32 weeks (HR = 2.38, 95% CI 1.26–4.49). In sensitivity analyses excluding women with diabetes and those with pre-eclampsia in the first pregnancy, results were similar (data not shown). Adjustment of the gestation analyses for birthweight did not substantially alter the results. In separate analyses for in-situ breast cancers (Additional file 1: Table S1) there was no evidence of raised risk for either women with high birthweight first-born or short gestation births, although this was based on much smaller numbers than for invasive tumours.

**Table 2:** Invasive breast cancer risk by birthweight and gestation of first-born singleton offspring by menopausal status^a^

Risk factor	Pre-menopausal^a^			Post-menopausal^a^			Breast cancer overall		
	No. of cases	HR^b^	95% CI	No. of cases	HR^b^	95% CI	No. of cases	HR^b^	95% CI
Birthweight (g)									
< 2000	8	2.05	1.01–4.17^c^	10	0.91	0.48-1.70	18	1.21	0.76–1.94
2000–2499	11	0.78	0.42–1.43	56	1.10	0.83–1.45	67	1.03	0.80–1.33
2500–2999	62	1.06	0.79–1.42	231	1.13	0.97–1.32	293	1.12	0.97–1.28
3000–3499	162	1.00		514	1.00		676	1.00	
3500–3999	141	1.10	0.88–1.38	360	0.98	0.85–1.12	501	1.01	0.90–1.13
4000–4499	35	1.01	0.70–1.46	86	1.01	0.80–1.27	121	1.01	0.83–1.22
≥ 4500	10	1.73	0.91–3.28	20	1.46	0.93–2.28	30	1.53	1.06–2.21^c^
Not known	20	1.76	1.11–2.81^c^	41	0.98	0.71–1.35	61	1.14	0.88–1.49
*p* trend^d^		0.55			0.50			0.86	
*p* interaction (trend) = 0.39									
Gestation (weeks)									
26–31	10	2.38	1.26–4.49^e^	12	0.95	0.53–1.68	22	1.30	0.85–1.99
32–36	33	1.23	0.85–1.78	117	1.09	0.89–1.33	150	1.10	0.92–1.32
37–39	116	0.92	0.73–1.15	355	1.14	0.99–1.30	471	1.07	0.95–1.21
40–41	200	1.00		481	1.00		681	1.00	
≥ 42	70	0.93	0.71–1.22	175	1.06	0.89–1.26	245	1.02	0.88–1.18
Not known	20	0.83	0.53–1.32	178	1.09	0.91–1.30	198	1.04	0.88–1.22
*p* trend^d^		0.03			0.28			0.05	
*p* interaction (trend) = 0.21									

*HR* hazard ratio, *CI* confidence interval

^a^Menopausal status at breast cancer incidence

^b^Adjusted for: attained age (Cox regression time scale); time since recruitment to cohort (0, 1–2, 3+ years); birth cohort (1908–1939, 1940–1949, 1950–1959, 1960–1969, 1970–1996); benign breast disease (yes, no); family history of breast cancer in first-degree relatives (yes, no); socio-economic score (ACORN score as trend, missing); own birthweight (trend, missing); age at menarche (trend, missing); parity (trend); age at first pregnancy (trend); cumulative duration of breast feeding (none, duration trend when reported); current oral contraceptive use before menopause (yes, no); height at age 20 (trend, missing); alcohol consumption (never regular, trend current drinker 1 to < 60 g/day, current drinker 60+ g/day, past drinker, drinker with unknown details); age started smoking (never, < 17, 17–19, 20+, missing); physical activity (log(metabolic equivalent) trend, missing); pre-menopausal body mass index at age 20 years (trend, missing); menopausal status (pre- or post-menopausal), and for those post-menopausal, post-menopausal body mass index (trend, missing), menopausal hormone therapy use (never used, ex-user, current oestrogen-only user, current oestrogen plus progestogen user, current user of other types, missing), and age at menopause (trend, missing)

^c^*p* < 0.05

^d^Excluding not known category

^e^*p* < 0.01

The oestrogen receptor (ER) status of the tumour was known for 99.3% of invasive breast cancers. The raised risk in mothers of high birthweight first-borns appeared present for both ER-positive and ER-negative tumours, although it was not significant in either, whereas the raised risk for mothers of low gestation first-borns appeared evident only for ER-positive tumours, although again this was not significant (Table 3).

**Table 3:** Invasive breast cancer risk by birthweight and gestation of first-born singleton offspring by oestrogen-receptor status^a^

	Oestrogen receptor status^a^					
	Positive			Negative		
	No. of cases	HR^b^	95% CI	No. of cases	HR^b^	95% CI
Birthweight (g)						
< 2000	16	1.32	0.80–2.17	2	0.86	0.21–3.49
2000–2499	58	1.09	0.83–1.42	7	0.70	0.33–1.51
2500–2999	228	1.06	0.90–1.23	57	1.42	1.03–1.96^c^
3000–3499	555	1.00		104	1.00	
3500–3999	404	0.99	0.87–1.13	81	1.05	0.79–1.41
4000–4499	95	0.97	0.78–1.21	20	1.07	0.66–1.73
≥ 4500	22	1.38	0.89–2.12	6	1.98	0.87–4.51
Not known	52	1.19	0.89–1.58	8	1.01	0.49–2.07
*p* trend^d^		*0.43*			*0.09*	
*p* interaction (trend) = 0.06						
Gestation (weeks)						
26–31	19	1.31	0.82–2.08	3	0.97	0.31–3.06
32–36	123	1.05	0.85–1.29	23	0.95	0.60–1.52
37–39	377	0.93	0.82–1.06	81	0.91	0.68–1.21
40–41	543	1.00		115	1.00	
42–49	199	0.96	0.81–1.15	38	0.84	0.57–1.24
Not known	169	1.02	0.85–1.22	25	0.76	0.48–1.20
*p* trend^d^		*0.06*			*0.55*	
*p* interaction (trend) = 0.82						

*HR* hazard ratio, *CI* confidence interval

^a^Oestrogen-receptor status of breast cancer

^b^Adjusted for: attained age (Cox regression time scale); time since recruitment to cohort (0, 1–2, 3+ years); birth cohort (1908–1939, 1940–1949, 1950–1959, 1960–1969, 1970–1996); benign breast disease (yes, no); family history of breast cancer in first-degree relatives (yes, no); socio-economic score (ACORN score as trend, missing); own birthweight (trend, missing); age at menarche (trend, missing); parity (trend); age at first pregnancy (trend); cumulative duration of breast feeding (none, duration trend when reported); current oral contraceptive use before menopause (yes, no); height at age 20 (trend, missing); alcohol consumption (never regular, trend current drinker 1 to < 60 g/day, current drinker 60+ g/day, past drinker, drinker with unknown details); age started smoking (never, < 17, 17–19, 20+, missing); physical activity (log(metabolic equivalent) trend, missing); pre-menopausal body mass index at age 20 years (trend, missing); menopausal status (pre- or post-menopausal), and for those post-menopausal, post-menopausal body mass index (trend, missing), menopausal hormone therapy use (never used, ex-user, current oestrogen-only user, current oestrogen plus progestogen user, current user of other types, missing), and age at menopause (trend, missing)

^c^*p* < 0.05

^d^Excluding not known category

When we divided follow-up time into < 15 years and ≥ 15 years since first delivery (Table 4), the effect of high birthweight was significant for the latter period, whereas the trend with gestation appeared steeper in the first 15 years of follow-up, although not significantly based on relatively small numbers.

**Table 4:** Invasive breast cancer risk by birthweight and gestation of firstborn singleton offspring by duration post-delivery

	Time since first delivery					
	< 15 years			≥15 years		
	No. of cases	HR^a^	95% CI	No. of cases	HR^a^	95% CI
Birthweight (g)						
< 2000	2	0.87	0.21–3.54	16	1.27	0.77–2.09
2000–2499	6	0.80	0.35–1.83	61	1.06	0.82–1.38
2500–2999	22	0.78	0.49–1.26	271	1.16	1.00–1.34^b^
3000–3499	79	1.00		597	1.00	
3500–3999	62	0.95	0.68–1.32	439	1.02	0.90–1.15
4000–4499	18	0.94	0.56–1.58	103	1.01	0.82–1.25
≥ 4500	5	1.37	0.55–3.39	25	1.56	1.04–2.33^b^
Not known	8	1.48	0.72–3.07	53	1.11	0.83–1.47
*p* trend^c^		*0.42*			*0.53*	
*p* interaction (trend) = 0.32						
Gestation (weeks)						
26–31	4	1.93	0.71–5.27	18	1.21	0.75–1.94
32–36	17	1.30	0.78–2.19	133	1.09	0.90–1.32
37–39	46	0.73	0.51–1.04	425	1.13	1.00–1.28
40–41	94	1.00		587	1.00	
≥ 42	33	0.95	0.64–1.42	212	1.03	0.88–1.20
Not known	8	0.84	0.41–1.73	190	1.06	0.90–1.25
*p* trend^c^		*0.32*			*0.09*	
*p* interaction (trend) = 0.72						

*HR* hazard ratio, *CI* confidence interval

^a^Adjusted for: attained age (Cox regression time scale); time since recruitment to cohort (0, 1–2, 3+ years); birth cohort (1908–1939, 1940–1949, 1950–1959, 1960–1969, 1970–1996); benign breast disease (yes, no); family history of breast cancer in first-degree relatives (yes, no); socio-economic score (ACORN score as trend, missing); own birthweight (trend, missing); age at menarche (trend, missing); parity (trend); age at first pregnancy (trend); cumulative duration of breast feeding (none, duration trend when reported); current oral contraceptive use before menopause (yes, no); height at age 20 (trend, missing); alcohol consumption (never regular, trend current drinker 1 to < 60 g/day, current drinker 60+ g/day, past drinker, drinker with unknown details); age started smoking (never, < 17, 17–19, 20+, missing); physical activity (log(metabolic equivalent) trend, missing); pre-menopausal body mass index at age 20 years (trend, missing); menopausal status (pre- or post-menopausal), and for those post-menopausal, post-menopausal body mass index (trend, missing), menopausal hormone therapy use (never used, ex-user, current oestrogen-only user, current oestrogen plus progestogen user, current user of other types, missing), and age at menopause (trend, missing)

^b^*p* < 0.05

^c^Excluding not known category

When analysed separately for first deliveries under the age of 30 years and older than this, the effects of birthweight and gestation were in the same direction for each, although not significantly based on smaller numbers (Additional file 1: Table S2).

Analyses of risk in relation to most recent pregnancy rather than first pregnancy gave less marked results (Table 5). Risk was non-significantly raised for mothers of high birthweight offspring and appeared to be unrelated to gestation of offspring. Repetition of the above analyses without adjustment for confounders (other than age) (Additional file 1: Tables S3–S6) gave very similar results—HRs for high birthweight tended to be slightly more elevated, and those for low birthweight slightly less elevated, than in the adjusted analyses. Repetition of the adjusted gestation analyses with and without adjustment for breast feeding (since breast feeding is associated with breast tissue differentiation) showed no significant interaction (*p* = 0.31).

**Table 5:** Invasive breast cancer risk by birthweight and gestation of most-recent singleton birth^a^ by menopausal status^b^

	Premenopausal^b^			Postmenopausal^b^			Breast cancer overall		
	No. of cases	HR^c^	95% CI	No. of cases	HR^c^	95% CI	No. of cases	HR^c^	95% CI
Birthweight (g)									
< 2000	4	1.25	0.46–3.37	6	0.72	0.32–1.61	10	0.87	0.47–1.63
2000–2499	11	1.10	0.60–2.03	28	0.84	0.57–1.23	39	0.90	0.65–1.24
2500–2999	29	0.62	0.42–0.92^d^	147	0.99	0.82–1.19	176	0.90	0.76–1.07
3000–3499	158	1.00		470	1.00		628	1.00	
3500–3999	155	0.95	0.76–1.19	415	0.96	0.84–1.09	570	0.96	0.85–1.07
4000–4499	60	1.06	0.78–1.42	137	0.99	0.81–1.19	197	1.01	0.86–1.18
≥ 4500	12	0.94	0.52–1.70	42	1.33	0.97–1.83	54	1.22	0.92–1.61
Not known	18	1.09	0.63–1.89	43	1.05	0.76–1.43	57	1.05	0.80–1.38
*p* trend^e^		*0.18*			*0.36*			*0.15*	
*p* interaction (trend) = 0.49									
Gestation (weeks)									
26–31	2	0.73	0.18–2.94	10	1.08	0.58–2.02	12	1.00	0.56–1.77
32–36	27	1.22	0.82–1.83	112	1.03	0.84–1.27	139	1.06	0.89–1.28
37–39	131	0.99	0.79–1.24	340	0.98	0.85–1.12	471	0.98	0.87–1.10
40–41	195	1.00		521	1.00		716	1.00	
≥ 42	64	1.23	0.93–1.64	113	0.94	0.77–1.15	177	1.03	0.87–1.21
Not known	24	0.93	0.60–1.42	192	1.04	0.88–1.23	216	1.03	0.88–1.20
*p* trend^e^		*0.99*			*0.52*			*0.59*	
*p* interaction (trend) = 0.74									

*HR* hazard ratio, *CI* confidence interval

^a^Women are censored (removed from further analytic follow-up) upon reaching a twin pregnancy

^b^Menopausal status at breast cancer incidence

^c^Adjusted for: attained age (Cox regression time scale); time since recruitment to cohort (0, 1–2, 3+ years); birth cohort (1908–1939, 1940–1949, 1950–1959, 1960–1969, 1970–1996); benign breast disease (yes, no); family history of breast cancer in first-degree relatives (yes, no); socio-economic score (ACORN score as trend, missing); own birthweight (trend, missing); age at menarche (trend, missing); parity (trend); age at first pregnancy (trend); cumulative duration of breast feeding (none, duration trend when reported); current oral contraceptive use before menopause (yes, no); height at age 20 (trend, missing); alcohol consumption (never regular, trend current drinker 1 to < 60 g/day, current drinker 60+ g/day, past drinker, drinker with unknown details); age started smoking (never, < 17, 17–19, 20+, missing); physical activity (log(metabolic equivalent) trend, missing); pre-menopausal body mass index at age 20 years (trend, missing); menopausal status (pre- or post-menopausal), and for those post-menopausal, post-menopausal body mass index (trend, missing), menopausal hormone therapy use (never used, ex-user, current oestrogen-only user, current oestrogen plus progestogen user, current user of other types, missing), and age at menopause (trend, missing)

^d^*p* < 0.05

^e^Excluding not known category

## Discussion

Mammary cells in animals proliferate in the first and second trimesters of pregnancy and differentiate in the third trimester [45], and the same is seen in the breast in humans [46, 47]. Thus, one might expect that pregnancies that continue to term or beyond might increase the extent of differentiation and hence protect (in the long term) against breast cancer. Conversely, a pregnancy that ends at an early stage might lead to increased risk through cell proliferation and high sex hormone levels promoting growth of partially transformed cells [36], without subsequent differentiation. Our results were in accord with this, with the risk greatest for early gestation births and decreasing with longer gestation, significantly for pre-menopausal tumours and borderline significantly for breast cancer overall. Previous analyses of the relation of long-term risk to gestation of the first birth have been based on record linkage; the results have not been entirely consistent and also give some uncertainty in comparison because of varying categorisations of gestation. Most have found results in the same direction as ours, significantly [15, 17, 18, 22] or not significantly [21], but some have not found such a relation [19, 20, 34]. Studies have generally found induced abortion unrelated to risk of subsequent breast cancer [48], but abortion is generally at far shorter gestations than those analysed in our data; we analysed only deliveries at ≥ 26 weeks. Our analyses excluded births of twins, and in sensitivity analyses were not materially altered by exclusion of pre-eclamptic births, but we cannot exclude the possibility that other indications for early delivery were responsible for the raised risk after short gestation births.

The effect of gestation appeared to be greater for pre-menopausal than for post-menopausal breast cancer. No previous study has published analyses by menopausal status, but in several studies all, or almost all, cases were incident at ages under 50 years [18–20, 33, 34], or separate analyses were conducted for ages < 40 years [17, 22], and therefore presumably largely pre-menopausal. Two studies found raised risks for short gestations in the presumed largely pre-menopausal group [17, 18], but several did not [19, 20, 22, 34]. In one investigation, oestradiol levels in early pregnancy have been found to be associated with a raised risk of subsequent breast cancer at young ages [49], but in another they were not [50], and in general risks in such studies have been inconsistent and unreplicated [49, 50].

In our data, the effect of short gestation pregnancies was somewhat greater, but not significantly so, in the first 15 years after delivery. A potential reason is the higher oestrogen levels in pre-term than longer pregnancies [51, 52], which might then promote the growth of already transformed cells in the years soon after. The only previous analyses dividing at 15 years after delivery found no effect of short gestation before or after 15 years [21], or an effect peaking at 10–14 years [22].

Fewer previous studies have analysed the risk of breast cancer in relation to birthweight of the offspring than have analysed gestation. We found the risk was raised significantly after a high birthweight first-born and to a lesser extent also after the lowest birthweights. Most other studies have also found the risk raised after a high birthweight first-born [7, 19–21, 23], although one found the opposite [18] and some reported no relation based on smaller numbers [16, 53]. Published risks have not been raised, however, for women with low birthweight offspring.

Women giving birth to heavier offspring tend to have higher oestrogen and free oestrogen levels [8, 9, 12–14] and insulin-like growth factor 1 (IGF1) levels [10] in pregnancy, giving a plausible mechanism for an association of high birthweight of offspring with maternal breast cancer risk.

The association of gestation with breast cancer risk was similar for ER-positive tumours to that for breast cancer overall (although not significantly, based on smaller numbers), but an association was not seen for ER-negative cancer. There do not appear to be any previous analyses by hormone receptor status with which to compare this. For high birthweight, the effect was greater, but not significantly so, for ER-negative tumours, in accord with the only previous, also not significant, analysis [23]. The effects of birthweight and gestation appeared not to be present, albeit based on smaller numbers, for in-situ rather than invasive tumours. To our knowledge there have been no previous analyses of risks of in-situ tumours.

While hormonal and histological explanations for the relation of gestation and birthweight to breast cancer risk seem the most obvious, it is also possible that maternal genetic susceptibility loci affecting offspring birthweight [54] or gestation [55] may affect breast cancer risk.

Our analyses of risk in relation to gestation and birthweight of most recent birth found no clear relations. This accords with the general tendency for first birth (e.g. age at this birth) to have a much greater impact on breast cancer risk than do subsequent births. The only previous analysis to provide data comparing breast cancer risk in relation to first birth and last birth found no significant effect of birthweight or gestation at either [20].

The previous literature on breast cancer risks in relation to gestation and birthweight of the offspring has, with two exceptions based on small numbers [7, 53], been based on record linkage. This has the great strengths of using recorded data on pregnancy variables, and in certain instances of very large numbers of cases [15, 20, 21, 23, 24], but also certain disadvantages which make the data not entirely comparable with ours. First, these studies had little [19, 33] or no [15, 17, 18, 20–24, 34] ability to adjust their risk estimates for potential non-reproductive confounders such as maternal height, birthweight, alcohol, and smoking, nor for an important reproductive-related confounder, breast feeding [56]. Secondly, in several instances [16, 17, 21, 53], the birth analysed was the first within the period of recorded data covered in the study, rather than the first in the woman’s reproductive history. Thirdly, while the Scandinavian studies [15–17, 19, 22–24] were based on national follow-up, the large record-linkage studies from the US included only women whose birth and subsequent cancer had both occurred in the same state [18, 20, 21], with a potential for bias.

The quality of birth certificate data on birthweight tends to be high [57, 58], but data quality is also high in recall by mothers [59]. Overall, our analyses based on a recruited epidemiological cohort, and those previously based on record linkage, have different strengths and weaknesses; hence, when they find similar results, this strengthens the case that these are real effects, not artefacts. For the effects of short gestation and high birthweight, our results are similar to those from the preponderance of record linkage studies and, with one exception for gestation [19] and one for birthweight [16], to all those from record linkage cohort studies.

## Conclusions

Our data add to evidence that a short gestation compared with a longer gestation first pregnancy results in increased breast cancer risk, and suggest that this increase occurs in pre-menopausal but not post-menopausal women. A potential mechanism is that short gestation pregnancies may result in hormonal stimulation and breast proliferation early in pregnancy, without the opportunity for differentiation occurring later in pregnancy. On birthweight, we found a raised risk for mothers of heavier first-borns, in accord with most previous studies, and potentially acting via the association of birthweight with oestrogen and IGF1 levels.

## Additional file

Additional file 1: **Table S1.** In-situ breast cancer risk by birthweight and gestation of first-born singleton offspring by menopausal status. **Table S2.** Invasive breast cancer risk by birthweight and gestation of first-born by age at first delivery. **Table S3.** Relative risks of invasive breast cancer in relation to birthweight and gestation of first-born singleton offspring by menopausal status at breast cancer incidence; analyses unadjusted for confounders other than age. **Table S4.** Relative risks of invasive breast cancer in relation to birthweight and gestation of first-born singleton offspring by oestrogen receptor status of breast cancer; analyses unadjusted for confounders other than age. **Table S5.** Relative risks of invasive breast cancer in relation to birthweight and gestation of first-born singleton offspring by duration since delivery; analyses unadjusted for confounders other than age. **Table S6.** Relative risks of invasive breast cancer in relation to birthweight and gestation of most recent singleton birth by menopausal status at breast cancer incidence; analyses unadjusted for confounders other than age. (DOCX 53 kb)

## Abbreviations

CI: Confidence interval
ER: Oestrogen receptor
HR: Hazard ratio
IGF1: Insulin-like growth factor 1
NHSCR: National Health Service Central Register
