# Supplementary material for: Maternal breast cancer risk in relation to birthweight and gestation of her offspring
Source: Breast Cancer Res. 2018 Oct 5;20:110. doi: 10.1186/s13058-018-1035-6 (PMC6172803; doi:10.1186/s13058-018-1035-6)
Supplement: Supplementary file 1 — Table S1. In-situ breast cancer risk by birthweight and gestation of first-born singleton offspring by menopausal status. Table S2. Invasive breast cancer risk by birthweight and gestation of first-born by age at first delivery. Table S3. Relative risks of invasive breast cancer in relation to birthweight and gestation of first-born singleton offspring by menopausal status at breast cancer incidence; analyses unadjusted for confounders other than age. Table S4. Relative risks of invasive breast cancer in relation to birthweight and gestation of first-born singleton offspring by oestrogen receptor status of breast cancer; analyses unadjusted for confounders other than age. Table S5. Relative risks of invasive breast cancer in relation to birthweight and gestation of first-born singleton offspring by duration since delivery; analyses unadjusted for confounders other than age. Table S6. Relative risks of invasive breast cancer in relation to birthweight and gestation of most recent singleton birth by menopausal status at breast cancer incidence; analyses unadjusted for confounders other than age. (DOCX 53 kb) [file 13058_2018_1035_MOESM1_ESM.docx]

| **Table S1:** In-situ breast cancer risk by birthweight and gestation of firstborn singleton offspring, by menopausal status^a^ | | | | | | | | | | | | |  |
| --- | --- | --- | --- | --- | --- | --- | --- | --- | --- | --- | --- | --- | --- |
|  |  | Premenopausal^a^ | | |  | Postmenopausal^a^ | | |  | Breast cancer overall | | |  |
| Risk factor |  | No. of cases | HR^b^ | 95% CI |  | No. of cases | HR^b^ | 95% CI |  | No. of cases | HR^b^ | 95% CI |  |
| Birthweight (g) |  |  |  |  |  |  |  |  |  |  |  |  |  |
| <2000 |  | 0 | 0.54 | 0.13–2.25 |  | 4 | 1.55 | 0.93–2.56 |  | 4 | 1.40 | 0.52–3.79 |  |
| 2000-2499 |  | 2 |  |  |  | 14 |  |  |  | 16 | 1.28 | 0.76–2.15 |  |
| 2500-2999 |  | 22 | 1.82 | 1.06–3.13 |  | 55 | 1.47 | 1.06–2.06^c^ |  | 77 | 1.56 | 1.17–2.07^d^ |  |
| 3000-3499 |  | 33 | 1.00 |  |  | 95 | 1.00 |  |  | 128 | 1.00 |  |  |
| 3500-3999 |  | 30 | 1.16 | 0.71–1.93 |  | 94 | 1.37 | 1.03–1.82^c^ |  | 124 | 1.31 | 1.02–1.69^c^ |  |
| 4000-4499 |  | 2 | 0.63 | 0.24–1.61 |  | 18 | 1.01 | 0.61–1.66 |  | 20 | 0.87 | 0.54–1.40 |  |
| ≥4500 |  | 3 |  |  |  | 1 | 0.53 | 0.19–1.44 |  | 4 | 1.07 | 0.40–2.92 |  |
| Not known |  | 1 | 0.43 | 0.06–3.11 |  | 4 |  |  |  | 5 | 0.50 | 0.20–1.23 |  |
| *P* trend^e^ |  |  | *0.49* |  |  |  | *0.49* |  |  |  | *0.19* |  |  |
|  |  |  |  | *P-interaction (trend) = 0.39* | | | |  |  |  |  |  |  |
| Gestation (weeks) |  |  |  |  |  |  |  |  |  |  |  |  |  |
| 26-31 |  | 0 | 0.66 | 0.24–1.86 |  | 1 | 0.75 | 0.47–1.20 |  | 1 | 0.26 | 0.04–1.84 |  |
| 32-36 |  | 4 |  |  |  | 20 |  |  |  | 24 | 0.80 | 0.52–1.24 |  |
| 37-39 |  | 24 | 1.00 | 0.60–1.67 |  | 79 | 1.07 | 0.81–1.43 |  | 103 | 1.06 | 0.82–1.36 |  |
| 40-41 |  | 38 | 1.00 |  |  | 114 | 1.00 |  |  | 152 | 1.00 |  |  |
| ≥42 |  | 20 | 1.39 | 0.81–2.38 |  | 39 | 0.97 | 0.67–1.39 |  | 59 | 1.08 | 0.80–1.46 |  |
| Not known |  | 7 | 1.48 | 0.66–3.32 |  | 32 | 0.87 | 0.58–1.29 |  | 39 | 0.94 | 0.66–1.35 |  |
| *P* trend^e^ |  |  | *0.15* |  |  |  | *0.65* |  |  |  | *0.28* |  |  |
|  |  |  |  | *P-interaction (trend) = 0.27* | | | |  |  |  |  |  |  |
| HR = hazard ratio; CI = confidence interval  ^a^ Menopausal status at breast cancer incidence  ^b^ Adjusted for: attained age (Cox regression time scale); time since recruitment to cohort (0, 1–2, 3+ years); birth cohort (1908–39, 1940–49, 1950–59, 1960–69, 1970–96); benign breast disease (yes, no); family history of breast cancer in 1st degree relatives (yes, no); socio-economic score (ACORN score as trend, missing); own birthweight (trend, missing); age at menarche (trend, missing); parity (trend); age at first pregnancy (trend); cumulative duration of breast feeding (none, duration trend when reported); current oral contraceptive use before menopause (yes, no); height at age 20 (trend, missing); alcohol consumption (never regular, trend current drinker 1– <60g/day, current drinker 60+g/day, past drinker, drinker with unknown details); age started smoking (never, <17, 17–19, 20+, missing); physical activity (log(metabolic equivalent) trend, missing ); pre-menopausal body mass index at age 20 years (trend, missing); menopausal status (pre- or post-menopausal), and for those post-menopausal, post-menopausal body mass index (trend, missing), menopausal hormone therapy use (never used, ex-user, current estrogen only user, current estrogen plus progestogen user, current user of other types, missing), and age at menopause (trend, missing).  ^c^ p<0.05  ^d^ p<0.01  ^e^ Excluding not known category   \| **Table S2:** Invasive breast cancer risk by birthweight and gestation of firstborn^a^,  by age at first delivery \| \| \| \| \| \| \| \| \| \| \| \| \| \| \| \| --- \| --- \| --- \| --- \| --- \| --- \| --- \| --- \| --- \| --- \| --- \| --- \| --- \| --- \| --- \| \|  \|  \|  \| Age at 1^st^ delivery^a^ \| \| \| \| \| \| \| \| \|  \|  \|  \| <30 years \| \| \| \|  \| 30+ years \| \| \| \|  \| \|  \| No. of cases \| HR^b^ \| 95% CI \| \|  \| No. of cases \| HR^b^ \| 95% CI \|  \| \| \|  \| \| Birthweight (g) \| \|  \|  \|  \| \|  \|  \|  \|  \|  \|  \| \| \|  \| \|  \| <2000 \|  \| 14 \| 1.45 \| 0.85–2.47 \| \|  \| 4 \| 0.84 \| 0.31–2.27 \|  \| \|  \| \| \|  \| 2000-2499 \|  \| 48 \| 1.07 \| 0.80–1.44 \| \|  \| 17 \| 0.91 \| 0.55–1.51 \|  \| \|  \| \| \|  \| 2500-2999 \|  \| 214 \| 1.12 \| 0.95–1.31 \| \|  \| 71 \| 1.10 \| 0.83–1.45 \|  \| \|  \| \| \|  \| 3000-3499 \|  \| 494 \| 1.00 \|  \| \|  \| 165 \| 1.00 \|  \|  \| \|  \| \| \|  \| 3500-3999 \|  \| 343 \| 0.97 \| 0.84–1.11 \| \|  \| 142 \| 1.11 \| 0.89–1.39 \|  \| \|  \| \| \|  \| 4000-4499 \|  \| 77 \| 0.92 \| 0.73–1.18 \| \|  \| 38 \| 1.15 \| 0.80–1.63 \|  \| \|  \| \| \|  \| ≥4500 \|  \| 18 \| 1.43 \| 0.89–2.29 \| \|  \| 10 \| 1.58 \| 0.83–2.99 \|  \| \| \|  \| \|  \| Not known \|  \| 37 \| 1.01 \| 0.72–1.41 \| \|  \| 23 \| 1.53 \| 0.99–2.36 \|  \| \| \|  \| \|  \| *P* trend^c^ \|  \|  \| *0.19* \| \|  \|  \|  \| *0.25* \|  \|  \| \| \|  \| \|  \|  \|  \|  \| *P-interaction (trend) = 0.09* \| \| \| \| \| \|  \|  \| \| \|  \| \|  \|  \|  \|  \|  \| \|  \|  \|  \|  \|  \|  \| \| \|  \| \| Gestation (weeks) \| \|  \|  \|  \| \|  \|  \|  \|  \|  \|  \| \| \|  \| \|  \| 26-31 \|  \| 13 \| 1.19 \| \| 0.68–2.06 \|  \| 9 \| 1.65 \| 0.85–3.23 \|  \| \| \|  \| \|  \| 32-36 \|  \| 107 \| 1.15 \| \| 0.93–1.42 \|  \| 39 \| 1.03 \| 0.73–1.45 \|  \| \| \|  \| \|  \| 37-39 \|  \| 330 \| 1.12 \| \| 0.97–1.29 \|  \| 128 \| 0.98 \| 0.78–1.22 \|  \| \| \|  \| \|  \| 40-41 \|  \| 465 \| 1.00 \| \|  \|  \| 193 \| 1.00 \|  \|  \| \| \|  \| \|  \| ≥42 \|  \| 173 \| 1.02 \| \| 0.86–1.22 \|  \| 64 \| 1.02 \| 0.76–1.35 \|  \| \| \|  \| \|  \| Not known \|  \| 157 \| 1.10 \| \| 0.92–1.33 \|  \| 37 \| 0.90 \| 0.63–1.29 \|  \| \| \|  \| \|  \| *P* trend^c^ \|  \|  \| *0.06* \| \|  \|  \|  \| *0.47* \|  \|  \| \| \|  \| \|  \|  \|  \|  \| *P-interaction (trend) = 0.71* \| \| \| \| \| \|  \|  \| \| \|  \| \|  \| \| \| \| \| \| \| \| \| \| \| \|  \| \| \|   HR: Hazard Ratio; CI: Confidence Interval  ^a^ Singleton offspring  ^b^ Adjusted for: attained age (Cox regression time scale); time since recruitment to cohort (0, 1–2, 3+ years); birth cohort (1908–39, 1940–49, 1950–59, 1960–69, 1970–96); benign breast disease (yes, no); family history of breast cancer in 1st degree relatives (yes, no); socio-economic score (ACORN score as trend, missing); own birthweight (trend, missing); age at menarche (trend, missing); parity (trend); age at first pregnancy (trend); cumulative duration of breast feeding (none, duration trend when reported); current oral contraceptive use before menopause (yes, no); height at age 20 (trend, missing); alcohol consumption (never regular, trend current drinker 1– <60g/day, current drinker 60+g/day, past drinker, drinker with unknown details); age started smoking (never, <17, 17–19, 20+, missing); physical activity (log(metabolic equivalent) trend, missing ); pre-menopausal body mass index at age 20 years (trend, missing); menopausal status (pre- or post-menopausal), and for those post-menopausal, post-menopausal body mass index (trend, missing), menopausal hormone therapy use (never used, ex-user, current estrogen only user, current estrogen plus progestogen user, current user of other types, missing), and age at menopause (trend, missing)  ^c^ Excluding not known category | | | | | | | | | | | | | |

| **Table S3:** Relative risks of invasive breast cancer in relation to birthweight and gestation of first born singleton offspring, by menopausal status at breast cancer incidence; analyses unadjusted for confounders, other than age | | | | | | | | | | | | |  |
| --- | --- | --- | --- | --- | --- | --- | --- | --- | --- | --- | --- | --- | --- |
|  |  | Premenopausal | | |  | Postmenopausal | | |  | Breast cancer overall | | |  |
| Risk factor |  | No. of cases | HR^a^ | 95% CI |  | No. of cases | HR^a^ | 95% CI |  | No. of cases | HR^a^ | 95% CI |  |
| Birthweight (g) |  |  |  |  |  |  |  |  |  |  |  |  |  |
| <2000 |  | 8 | 2.06 | 1.01-4.19^b^ |  | 10 | 0.95 | 0.51-1.78 |  | 18 | 1.25 | 0.78-2.00 |  |
| 2000-2499 |  | 11 | 0.78 | 0.42–1.43 |  | 56 | 1.09 | 0.83–1.44 |  | 67 | 1.02 | 0.80–1.31 |  |
| 2500-2999 |  | 62 | 1.04 | 0.78–1.39 |  | 231 | 1.10 | 0.94–1.29 |  | 293 | 1.09 | 0.95–1.25 |  |
| 3000-3499 |  | 162 | 1.00 |  |  | 514 | 1.00 |  |  | 676 | 1.00 |  |  |
| 3500-3999 |  | 141 | 1.12 | 0.89–1.41 |  | 360 | 1.01 | 0.88–1.15 |  | 501 | 1.04 | 0.92–1.16 |  |
| 4000-4499 |  | 35 | 1.07 | 0.74–1.54 |  | 86 | 1.07 | 0.85–1.35 |  | 121 | 1.07 | 0.88–1.29 |  |
| ≥4500 |  | 10 | 1.87 | 0.99–3.55 |  | 20 | 1.62 | 1.04–2.53^b^ |  | 30 | 1.69 | 1.17–2.24^c^ |  |
| Not known |  | 20 | 1.74 | 1.09–2.77^b^ |  | 41 | 0.98 | 0.72–1.35 |  | 61 | 1.15 | 0.88–1.49 |  |
| *P* trend^d^ |  |  | *0.31* |  |  |  | *0.88* |  |  |  | *0.21* |  |  |
|  |  |  | *P-interaction (trend) = 0.43* | | | | |  |  |  |  |  |  |
| Gestation (weeks) |  |  |  |  |  |  |  |  |  |  |  |  |  |
| 26-31 |  | 10 | 2.29 | 1.21–4.32^b^ |  | 12 | 0.95 | 0.53–1.68 |  | 22 | 1.29 | 0.84–1.97 |  |
| 32-36 |  | 33 | 1.22 | 0.84–1.76 |  | 117 | 1.07 | 0.87–1.31 |  | 150 | 1.09 | 0.91–1.30 |  |
| 37-39 |  | 116 | 0.93 | 0.74–1.17 |  | 355 | 1.13 | 0.99–1.30 |  | 471 | 1.07 | 0.95–1.21 |  |
| 40-41 |  | 200 | 1.00 |  |  | 481 | 1.00 |  |  | 681 | 1.00 |  |  |
| ≥42 |  | 70 | 0.91 | 0.70–1.20 |  | 175 | 1.07 | 0.90–1.27 |  | 245 | 1.02 | 0.88–1.18 |  |
| Not known |  | 20 | 0.83 | 0.52–1.31 |  | 178 | 1.05 | 0.88–1.25 |  | 198 | 1.01 | 0.86–1.19 |  |
| *P* trend^d^ |  |  | *0.02* |  |  |  | *0.38* |  |  |  | *0.07* |  |  |
|  |  |  | *P-interaction (trend) = 0.18* | | | | |  |  |  |  |  |  |
|  |  |  |  |  |  |  |  |  |  |  |  |  |  |
| HR = hazard ratio; CI = confidence interval  ^a^ Adjusted for: attained age (Cox regression time scale)  ^b^ p<0.05  ^c^ p<0.01  ^d^ Excluding not known category | | | | | | | | | | | | | |

| **Table S4**: Relative risks of invasive breast cancer in relation to birthweight and gestation of first born singleton offspring, by estrogen receptor status of breast cancer: analyses unadjusted for confounders other than age | | | | | | | | | | | |
| --- | --- | --- | --- | --- | --- | --- | --- | --- | --- | --- | --- |
|  |  |  | Estrogen receptor status | | | | | | |  |  |
|  |  |  | Positive | | |  | Negative | | |  |  |
|  | |  | No. of cases | HR^a^ | 95% CI |  | No. of cases | HR^a^ | 95% CI |  |  |
| Birthweight (g) | |  |  |  |  |  |  |  |  |  |  |
|  | <2000 |  | 16 | 1.36 | 0.83–2.23 |  | 2 | 0.89 | 0.22–3.58 |  |  |
|  | <2500 |  | 58 | 1.08 | 0.82–1.41 |  | 7 | 0.70 | 0.32–1.50 |  |  |
|  | <3000 |  | 228 | 1.03 | 0.88–1.20 |  | 57 | 1.39 | 1.01–1.92^b^ |  |  |
|  | <3500 |  | 555 | 1.00 |  |  | 104 | 1.00 |  |  |  |
|  | <4000 |  | 404 | 1.02 | 0.90–1.16 |  | 81 | 1.08 | 0.81–1.45 |  |  |
|  | <4500 |  | 95 | 1.02 | 0.82–1.27 |  | 20 | 1.13 | 0.70–1.83 |  |  |
|  | <5000 |  | 22 | 1.52 | 0.99–2.33 |  | 6 | 2.17 | 0.95–4.93 |  |  |
|  | Not known |  | 52 | 1.18 | 0.89–1.58 |  | 8 | 1.01 | 0.49–2.07 |  |  |
|  | *P* trend^c^ |  |  | *0.09* |  |  |  | *0.19* |  |  |  |
|  |  |  |  | *P-interaction (trend) = 0.06* | | | | |  |  |  |
|  |  |  |  |  |  |  |  |  |  |  |  |
| Gestation (weeks) | |  |  |  |  |  |  |  |  |  |  |
|  | 26-31 |  | 19 | 1.29 | 0.82–2.05 |  | 3 | 0.96 | 0.30–3.03 |  |  |
|  | 32-36 |  | 123 | 1.04 | 0.84–1.27 |  | 23 | 0.94 | 0.59–1.51 |  |  |
|  | 37-39 |  | 377 | 0.93 | 0.82–1.06 |  | 81 | 0.91 | 0.68–1.20 |  |  |
|  | 40-41 |  | 543 | 1.00 |  |  | 115 | 1.00 |  |  |  |
|  | 42-49 |  | 199 | 0.97 | 0.82–1.15 |  | 38 | 0.85 | 0.58–1.25 |  |  |
|  | Not known |  | 169 | 0.99 | 0.83–1.19 |  | 25 | 0.74 | 0.47–1.17 |  |  |
|  | *P* trend^c^ |  |  | *0.08* |  |  |  | *0.58* |  |  |  |
|  |  |  |  | *P-interaction (trend) = 0.83* | | | | |  |  |  |
| HR = hazard ratio; CI = confidence interval  ^a^ Adjusted for: attained age (Cox regression time scale)  ^b^ p<0.05 | | | | | | | | | | |  |
| ^c^ Excluding not known category | | | | | | | | | | |  |

| **Table S5:** Relative risks of invasive breast cancer in relation to birthweight and gestation of first born singleton offspring, by duration since delivery : analyses unadjusted for confounders other than age | | | | | | | | | | | | |
| --- | --- | --- | --- | --- | --- | --- | --- | --- | --- | --- | --- | --- |
|  |  |  | Time since 1^st^ delivery | | | | | | | | | |
|  |  |  | <15 years | | |  | | ≥15 years | | | | |
|  | |  | No. of cases | HR^a^ | 95% CI |  | | No. of cases | | HR^a^ | 95% CI |  |
| Birthweight (g) | |  |  |  |  |  | |  | |  |  |  |
|  | <2000 |  | 2 | 0.89 | 0.22–3.60 |  | | 16 | | 1.31 | 0.80–2.15 |  |
|  | 2000-2499 |  | 6 | 0.78 | 0.34–1.80 |  | | 61 | | 1.05 | 0.81–1.37 |  |
|  | 2500-2999 |  | 22 | 0.76 | 0.48–1.23 |  | | 271 | | 1.13 | 0.98–1.30 |  |
|  | 3000-3499 |  | 79 | 1.00 |  |  | | 597 | | 1.00 |  |  |
|  | 3500-3999 |  | 62 | 0.96 | 0.69–1.34 |  | | 439 | | 1.05 | 0.93–1.18 |  |
|  | 4000-4499 |  | 18 | 0.98 | 0.59–1.64 |  | | 103 | | 1.08 | 0.87–1.33 |  |
|  | ≥4500 |  | 5 | 1.48 | 0.60–3.65 |  | | 25 | | 1.72 | 1.16–2.57^b^ |  |
|  | Not known |  | 8 | 1.43 | 0.69–2.96 |  | | 53 | | 1.11 | 0.84–1.48 |  |
|  | *P* trend^c^ |  |  | *0.28* |  |  | |  | | *0.78* |  |  |
|  |  |  |  | *P-interaction (trend) = 0.36* | | | | | | |  |  |
|  |  |  |  |  |  | |  | |  |  |  |  |
| Gestation (weeks) | |  |  |  |  | |  | |  |  |  |  |
|  | 26-31 |  | 4 | 1.92 | 0.71–5.22 | |  | | 18 | 1.20 | 0.75–1.92 |  |
|  | 32-36 |  | 17 | 1.29 | 0.77–2.17 | |  | | 133 | 1.07 | 0.89–1.30 |  |
|  | 37-39 |  | 46 | 0.74 | 0.52–1.05 | |  | | 425 | 1.13 | 0.99–1.28 |  |
|  | 40-41 |  | 94 | 1.00 |  | |  | | 587 | 1.00 |  |  |
|  | ≥42 |  | 33 | 0.95 | 0.64–1.41 | |  | | 212 | 1.04 | 0.88–1.21 |  |
|  | Not known |  | 8 | 0.85 | 0.41–1.75 | |  | | 190 | 1.03 | 0.87–1.21 |  |
|  | *P* trend^c^ |  |  | *0.32* |  | |  | |  | *0.12* |  |  |
|  |  |  |  | *P-interaction (trend) = 0.68* | | | | | | |  |  |
| HR = hazard ratio; CI = confidence interval  ^a^ Adjusted for: attained age (Cox regression time scale)  ^b^ p<0.01  ^c^ Excluding not know category | | | | | | | | | | | |  |

| **Table S6**: Relative risks of invasive breast cancer in relation to birthweight and gestation of most recent singleton birth^a^, by menopausal status at breast cancer incidence: analyses unadjusted for confounders other than age | | | | | | | | | | | | | | | |
| --- | --- | --- | --- | --- | --- | --- | --- | --- | --- | --- | --- | --- | --- | --- | --- |
|  | | |  |  |  |  |  |  |  |  | |  | | |  |
|  | | |  | Premenopausal | | |  | Postmenopausal | | |  | Breast cancer overall | | | |
|  | | |  | No. of cases | HR^b^ | 95% CI |  | No. of cases | HR^b^ | 95% CI |  | No. of cases | HR^b^ | 95% CI | |
| Birthweight (g) | | |  |  |  |  |  |  |  |  |  |  |  |  | |
|  | | <2000 |  | 4 | 1.34 | 0.50–3.61 |  | 6 | 0.76 | 0.34–1.70 |  | 10 | 0.92 | 0.49–1.72 | |
|  | | 2000-2499 |  | 11 | 1.12 | 0.61–2.05 |  | 28 | 0.85 | 0.58–1.24 |  | 39 | 0.91 | 0.66–1.25 | |
|  | | 2500-2999 |  | 29 | 0.61 | 0.41–0.91^c^ |  | 147 | 0.96 | 0.80–1.16 |  | 176 | 0.88 | 0.74–1.04 | |
|  | | 3000-3499 |  | 158 | 1.00 |  |  | 470 | 1.00 |  |  | 628 | 1.00 |  | |
|  | | 3500-3999 |  | 155 | 0.96 | 0.77–1.19 |  | 415 | 0.97 | 0.85–1.11 |  | 570 | 0.97 | 0.87–1.09 | |
|  | | 4000-4499 |  | 60 | 1.07 | 0.79–1.44 |  | 137 | 1.02 | 0.84–1.23 |  | 197 | 1.04 | 0.88–1.22 | |
|  | | ≥4500 |  | 12 | 0.96 | 0.53–1.73 |  | 42 | 1.42 | 1.03–1.94^c^ |  | 54 | 1.28 | 0.97–1.70 | |
|  | | Not known |  | 18 | 1.08 | 0.62–1.86 |  | 43 | 1.06 | 0.78–1.45 |  | 57 | 1.07 | 0.81–1.40 | |
|  | | *P* trend^d^ |  |  | *0.15* |  |  |  | *0.14* |  |  |  | *0.04* |  | |
|  | |  |  |  |  | *P-interaction (trend) = 0.63* | | | | |  |  |  |  | |
|  | |  |  |  |  |  |  |  |  |  |  |  |  |  | |
| Gestation (weeks) | | |  |  |  |  |  |  |  |  |  |  |  |  | |
|  | | 26-31 |  | 2 | 0.72 | 0.18–2.92 |  | 10 | 1.07 | 0.57–2.01 |  | 12 | 0.99 | 0.56–1.76 | |
|  | | 32-36 |  | 27 | 1.22 | 0.82–1.83 |  | 112 | 1.01 | 0.83–1.24 |  | 139 | 1.05 | 0.88–1.26 | |
|  | | 37-39 |  | 131 | 1.00 | 0.80–1.24 |  | 340 | 0.98 | 0.85–1.12 |  | 471 | 0.98 | 0.87–1.10 | |
|  | | 40-41 |  | 195 | 1.00 |  |  | 521 | 1.00 |  |  | 716 | 1.00 |  | |
|  | | ≥42 |  | 64 | 1.24 | 0.93–1.64 |  | 113 | 0.96 | 0.78–1.18 |  | 177 | 1.04 | 0.88–1.23 | |
|  | | Not known |  | 24 | 0.90 | 0.59–1.38 |  | 192 | 1.01 | 0.85–1.19 |  | 216 | 1.00 | 0.86–1.17 | |
|  | | *P* trend^d^ |  |  | *0.99* |  |  |  | *0.65* |  |  |  | *0.72* |  | |
|  | |  |  |  |  | *P-interaction (trend) = 0.82* | | | | |  |  |  |  | |
| HR = hazard ratio; CI = confidence interval  ^a^ Women are censored (removed from further analytic follow-up) upon reaching a twin pregnancy.  ^b^ Adjusted for: attained age (Cox regression time scale)  ^c^ p<0.05  ^d^ Excluding not known category | | | | | | | | | | | | | | | |
